# Usefulness of the delta neutrophil index to predict 30-day mortality in patients with ST segment elevation myocardial infarction

**DOI:** 10.1038/s41598-017-15878-5

**Published:** 2017-11-16

**Authors:** Taeyoung Kong, Tae Hoon Kim, Yoo Seok Park, Sung Phil Chung, Hye Sun Lee, Jung Hwa Hong, Jong Wook Lee, Je Sung You, Incheol Park

**Affiliations:** 1 Department of Emergency Medicine, Yonsei University College of Medicine, Seoul, Republic of Korea; 2 Division of Cardiology, Department of Internal Medicine, Yonsei University College of Medicine, Seoul, Republic of Korea; 3 Department of Research Affairs, Biostatistics Collaboration Unit, Yonsei University College of Medicine, Seoul, Republic of Korea; 4 Department of Health Insurance Research, National Health Insurance Service Ilsan Hospital, Gayang, Republic of Korea; 5 Department of Laboratory Medicine, Konyang University Hospital, Daejeon, Republic of Korea; 6 Research Institute of Bacterial Resistance, Yonsei University College of Medicine, Seoul, Korea

## Abstract

This study aimed to evaluate the association between the delta neutrophil index (DNI), which reflects immature granulocytes, and the severity of ST-elevation myocardial infarction (STEMI), as well as to determine the significance of the DNI as a prognostic marker for early mortality and other clinical outcomes in patients with STEMI who underwent reperfusion. This retrospective, observational cohort study was conducted using patients prospectively integrated in a critical pathway program for STEMI. We included 842 patients diagnosed with STEMI who underwent primary percutaneous coronary intervention (pPCI). Higher DNI values at time-I (within 2 h of pPCI; hazard ratio [HR], 1.075; 95% confidence interval [CI]: 1.046–1.108; p < 0.001) and time-24 (24 h after admission; HR, 1.066; 95% CI: 1.045–1.086; p < 0.001) were significant independent risk factors for 30-day mortality. Specifically, DNI values >2.5% at time-I (HR, 13.643; 95% CI: 8.13–22.897; p < 0.001) and > 2.9% at time-24 (HR, 12.752; 95% CI: 7.308–22.252; p < 0.001) associated with increased risks of 30-day mortality. In conclusion, an increased DNI value, which reflects the proportion of circulating immature granulocytes in the blood, was found to be an independent predictor of 30-day mortality and poor clinical outcomes in patients with acute STEMI post-pPCI.

## Introduction

Acute myocardial infarction (AMI) remains a major cause of mortality and morbidity worldwide, causing more than 150,000 deaths in the USA each year^1,2^. Despite recent improvements in critical care medicine, patients with ST-segment elevation myocardial infarction (STEMI) on the presenting electrocardiogram (ECG) remain at increased risk of mortality and serious morbidity if they survive the initial ischaemic event^3^. It is widely accepted that accurate and rapid assessment of the severity critically affects the treatment and prognosis of patients with STEMI^4^. Many studies have attempted to develop cardiac-specific markers or risk scoring systems to identify patients at increased risk and to provide prognostic information^5^. Recently, the roles of inflammatory markers for severity assessment in the early stage of STEMI have been attracting interest. In AMI, early ischaemic injury leads to an extreme inflammatory response^6^. Although primary percutaneous coronary intervention (pPCI) restores the patency of the epicardial coronary arteries, reperfusion injury by tissue oedema, endothelial disruption, and inflammation worsens ischaemia-related injury^7^. PCI itself is also a strong additional inflammatory stimulus and may cause acute systemic inflammatory responses, leading to post-PCI complications^8^.

Despite experimental and clinical evidence of the associations between inflammation and adverse outcomes, no specific inflammatory biomarkers are currently routinely used in the management of patients with STEMI^3,9^. The immature granulocyte is a practical marker of local and systemic inflammation^10–12^. The use of a specific automated blood cell analyser—a recent technological advancement—allows rapid determination of the delta neutrophil index (DNI), which reflects the fraction of circulating immature granulocytes in the blood, along with the complete blood count (CBC)^10,11,13–15^. Herein, we evaluated the significance of the DNI as a prognostic marker of early mortality in patients with STEMI who underwent pPCI. To the best of our knowledge, this is the first study to evaluate the association between the DNI and the severity of STEMI in the clinical setting.

## Results

**Figure 1:**
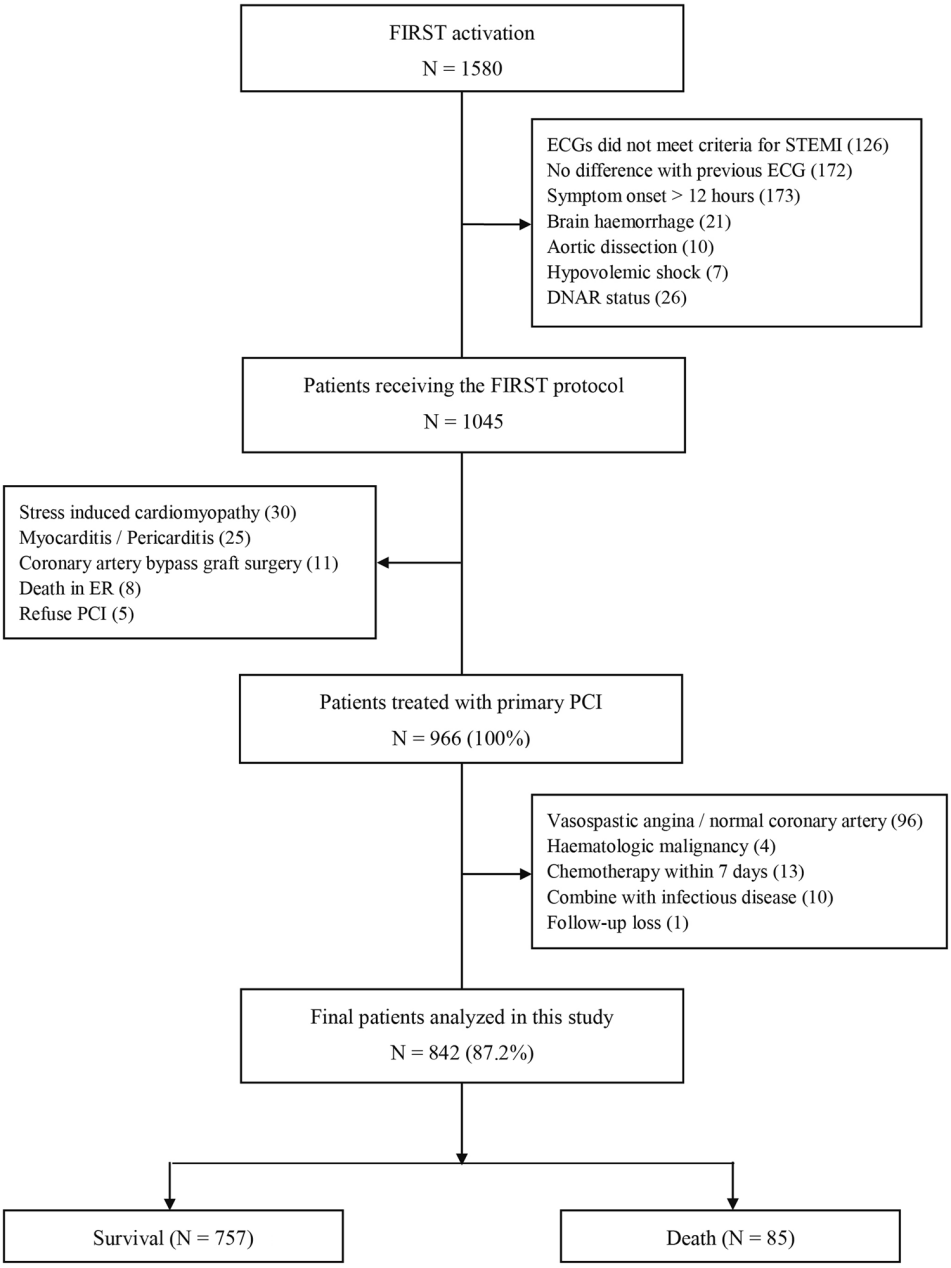
Flow diagram of patient enrolment. ECG, electrocardiogram; FIRST, Fast Interrogation Rule for ST-elevation Myocardial Infarction; DNAR, do not attempt resuscitation; PCI, percutaneous coronary intervention; STEMI, ST segment elevation myocardial infarction.

Figure 1 shows the enrolment and clinical outcome data for patients with STEMI registered in the Fast Interrogation Rule for ST-elevation Myocardial Infarction (FIRST) program. A total of 842 (87.2%) patients were enrolled in this study. Their baseline characteristics and clinical data are presented in Table 1. Of the 842 study patients, 85 patients expired within 30 days. Of these, 74 patients died from cardiac-related causes, including cardiogenic shock (n = 18), acute myocardial infarction (n = 19), heart failure (n = 16), sudden cardiac death (n = 15), malignant arrhythmia (n = 4), and left ventricular rupture (n = 2). On the other hand, 6 patients died from non-cardiac causes, including septic shock (n = 4), aortic dissection (n = 1), and cancer (n = 1), while the cause of death was unknown in 5 patients. The DNI for each patient was determined at time-0 (immediately on emergency department [ED] admission), time-I (within 2 h post-pPCI), and time-24 (24 h post-admission). The mean DNI values at time-I and time-24 were significantly higher in the non-survival group, i.e. among those who died within 30 days, than in the survival group (Table 1).

**Table 1:** Clinical characteristics of the study patients stratified according to 30-day mortality.

Variable	Total (N = 842)	30-day mortality		
		Survival (N = 757)	Death (N = 85)	*p-*value
Male sex (N, %)	667 (79.22)	610 (80.58)	57 (67.06)	0.004*
Body mass index (kg/m²)	24.20 ± 3.45	24.26 ± 3.41	23.69 ± 3.76	0.154
Use of LMWH (N, %)	18 (2.14)	17 (2.25)	1 (1.18)	0.999
Use of unfractionated heparin (N, %)	836 (99.29)	754 (99.60)	82 (96.47)	0.016*
GRACE score (point)	169.70 ± 46.75	163.47 ± 43.55	225.20 ± 36.64	<0.001*
Age (years)	63.39 ± 13.24	62.49 ± 13.18	71.42 ± 10.96	<0.001*
Heart rate (/min)	81.06 ± 26.41	80.98 ± 23.88	81.85 ± 43.04	0.855
SBP (mmHg)	126.57 ± 38.71	130.03 ± 35.60	95.68 ± 50.24	<0.001*
Creatinine (mg/dL)	1.24 ± 1.22	1.19 ± 1.14	1.74 ± 1.71	0.005*
Troponin-T (pg/mL)	673.02 ± 1650.60	536.57 ± 1366.20	1886.62 ± 2968.29	<0.001*
Arrest on admission (N, %)	22 (2.61)	10 (1.32)	12 (14.12)	<0.001*
Killip class				<0.001*
I (N, %)	380 (45.13)	369 (48.75)	11 (12.94)	
II (N, %)	164 (19.48)	159 (21.00)	5 (5.88)	
III (N, %)	125 (14.85)	101 (13.34)	24 (28.24)	
IV (N, %)	173 (20.55)	128 (16.91)	45 (52.94)	
**Clinical measurements**				
LVEF (%)	45.26 ± 12.31	46.98 ± 11.01	29.58 ± 12.52	<0.001*
NT-proBNP (pg/mL)	3123.6 ± 8133.8	2459.9 ± 7140.7	9029.6 ± 12815.3	<0.001*
Creatine kinase-MB (ng/mL)	28.53 ± 67.43	24.62 ± 58.07	63.32 ± 117.52	0.004*
White blood cell count (10^3/μL)	11.47 ± 4.03	11.36 ± 3.93	12.43 ± 4.74	0.049*
Neutrophil ratio (%)	65.04 ± 17.05	64.79 ± 16.74	67.28 ± 19.59	0.262
hs-CRP (mg/L)	25.10 ± 54.34	24.26 ± 54.82	33.19 ± 49.17	0.173
aPTT (s)	35.64 ± 28.79	35.00 ± 27.60	41.28 ± 37.47	0.137
Platelets (10^3/μL)	244.31 ± 81.84	246.15 ± 80.56	227.94 ± 91.33	0.052
Total cholesterol (mg/dL)	186.16 ± 47.58	187.99 ± 47.51	169.90 ± 45.29	<0.001*
Triglyceride (mg/dL)	115.63 ± 103.35	117.23 ± 106.26	97.88 ± 60.35	0.022*
Glucose (mg/dL)	196.38 ± 94.15	190.34 ± 88.76	250.26 ± 120.70	<0.001*
**Medical History**				
Hypertension (N, %)	434 (51.54)	388 (51.25)	46 (54.12)	0.617
Diabetes mellitus (N, %)	232 (27.59)	199 (26.32)	33 (38.82)	0.015*
COPD (N, %)	17 (2.02)	15 (1.98)	2 (2.35)	0.686
Hyperlipidaemia (N, %)	94 (11.16)	89 (11.76)	5 (5.88)	0.103
History of PCI (N, %)	106 (12.59)	96 (12.68)	10 (11.76)	0.809
CAOD (N, %)	145 (17.22)	124 (16.38)	21 (24.71)	0.054
Heart failure (N, %)	21 (2.49)	18 (2.38)	3 (3.53)	0.461
Arrhythmia (N, %)	20 (2.38)	19 (2.51)	1 (1.18)	0.711
Stroke (N, %)	43 (5.11)	31 (4.10)	12 (14.12)	<0.001*
PAOD (N, %)	11 (1.31)	9 (1.19)	2 (2.35)	0.307
Malignancy (N, %)	46 (5.46)	37 (4.89)	9 (10.59)	0.041*
Chronic kidney disease (N, %)	44 (5.23)	35 (4.62)	9 (10.59)	0.034*
Chronic liver disease (N, %)	7 (0.83)	6 (0.79)	1 (1.18)	0.527
**Procedural characteristics**				
Door-to-balloon time (min)	64.62 ± 32.55	63.39 ± 31.68	75.51 ± 37.99	0.006*
Procedure time (min)	40.50 ± 20.44	39.62 ± 19.97	48.31 ± 22.94	<0.001*
Type of contrast medium				0.217
Iopamidol (N, %)	198 (23.52)	177 (23.38)	21 (24.71)	
Iodixanol (N, %)	642 (76.25)	579 (76.49)	63 (74.12)	
Iopromide (N, %)	2 (0.24)	1 (0.13)	1 (1.18)	
Contrast volume (mL)	192.74 ± 76.01	193.32 ± 76.04	187.65 ± 76.01	0.515
Multivessel disease (N, %)	515 (61.16)	452 (59.71)	63 (74.12)	0.01*
LM involvement (N, %)	38 (4.51)	28 (3.70)	10 (11.76)	0.003*
DNI Time-0 (%)	0.78 ± 1.79	0.68 ± 1.58	1.66 ± 2.99	0.004*
DNI Time-I (%)	2.23 ± 5.24	1.30 ± 2.52	10.84 ± 11.96	<0.001*
DNI Time-24 (%)	2.47 ± 6.82	1.33 ± 2.58	15.65 ± 17.98	<0.001*

LMWH, low molecular weight heparin; GRACE, Global Registry of Acute Coronary Events; SBP, systolic blood pressure; LVEF, left ventricular ejection fraction; NT-proBNP, N-terminal pro-brain natriuretic peptide; hs-CRP, high-sensitivity C-reactive protein; aPTT, activated partial thromboplastin time; COPD, chronic obstructive pulmonary disease; PCI, Percutaneous Coronary Intervention; CAOD, coronary artery occlusive disease; PAOD, peripheral arterial occlusive disease; LM, left main coronary artery; DNI, delta neutrophil index. Data are expressed as the mean ± standard deviation or number (percentage). *P < 0.05.

**Figure 2:**
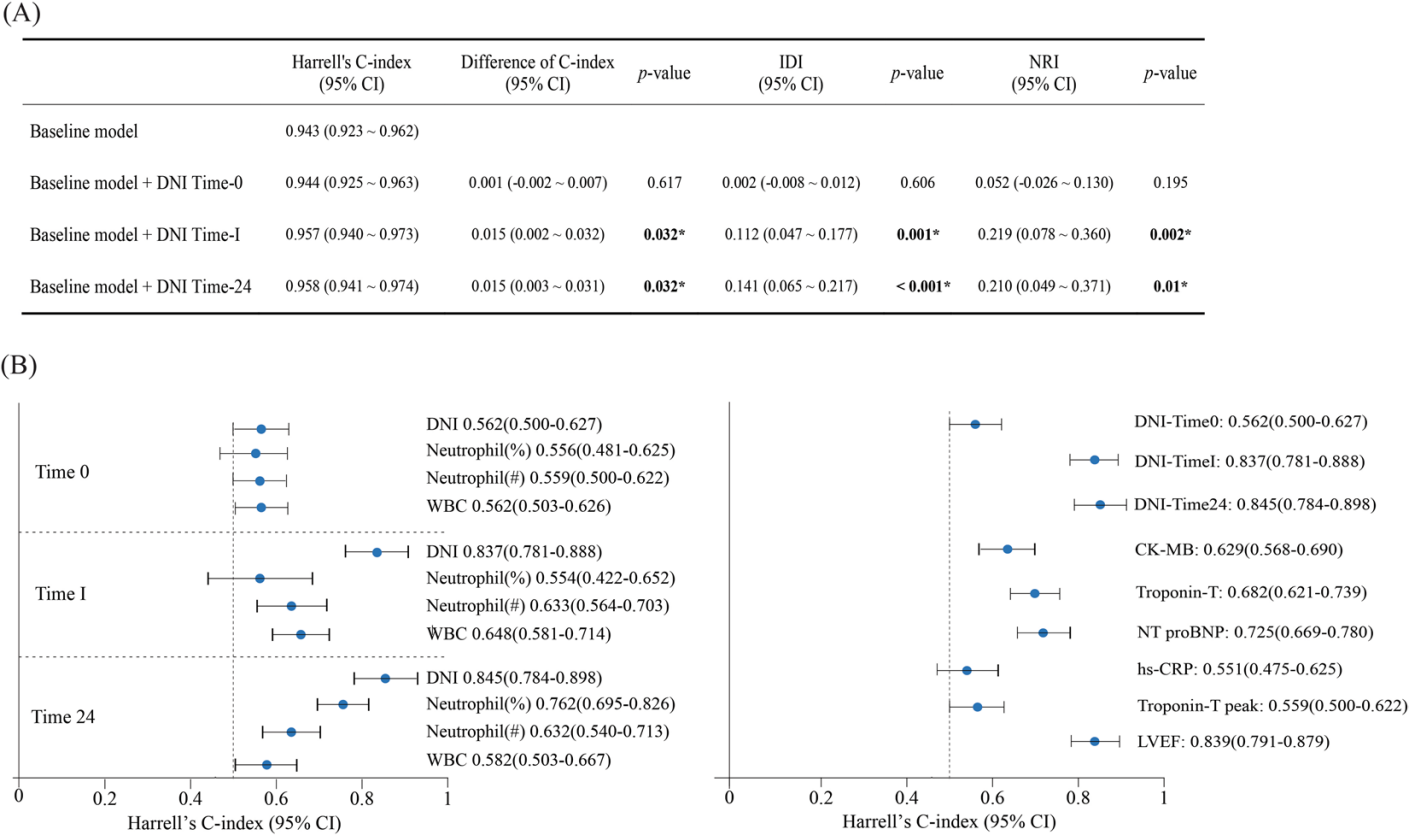
(**A**) Comparison of the performance of the survival models with and without the delta neutrophil index (DNI) by Harrell’s C-index, integrated discrimination improvement (IDI), and continuous net reclassification improvement (NRI). (**B**) Comparison of Harrell’s C-index for biomarkers at the time of emergency department admission, immediately after reperfusion, and 24 h after admission. Harrell’s C-index showed discriminative abilities for the risk stratification of 30-day mortality (statistical information in Supplement 3). CI, confidence interval; PCI, percutaneous coronary intervention; DNI, delta neutrophil index; WBC, white blood cell count; Neu(#), number of neutrophils; Neu(%), proportion of neutrophils; NT-proBNP, N-terminal pro-brain natriuretic peptide; Tn-T; Troponin-T; CK-MB, Creatinine kinase-MB.

The linear-mixed model revealed significant differences in the DNI values between patients grouped according to 30-day survival (group; p < 0.001, time; p < 0.001, group x time; p < 0.001). Further, significant differences in the DNI values were also seen between patients with and without development of heart failure (group; p < 0.001, time; p < 0.001, group x time; p < 0.001). Univariable Cox regression analysis confirmed these significant differences in the DNI values at times I and 24 between the non-survival and survival groups (Table 2). The multivariable Cox proportional hazard model further confirmed the associations between increased DNI values at times I and 24 and an increased risk of 30-day mortality among patients with STEMI who underwent pPCI (Table 3). Similarly, in both the univariable and multivariable logistic regression analyses, increased DNI values at times I and 24 were significantly associated with increased risks of heart failure in patients with acute STEMI after pPCI (Supplements 1 and 2). The DNI at time-I showed a moderately negative correlation with the left ventricular ejection fraction on discharge (r = −0.313, p < 0.001). To evaluate the predictability of the DNI over time, we calculated Harrell’s C-index using a baseline model based on the multivariable Cox proportional hazard analysis. The C index of each Cox model was assessed to evaluate its discriminatory usefulness. The C-statistics of models 1 (null model), 2 (null model + DNI time-0), 3 (null model + DNI time-I), and 4 (null model + DNI time-24) were 0.943, 0.944, 0.957, and 0.958, respectively. Despite relatively high C-statistics, adding the DNI over time to the survival models revealed only a tendency of increased C-statistics (Fig. 2A and Supplement 3). When comparing the C-statistics of the DNI to those of other markers, the C-statistics of the DNI at times I and 24 were statistically superior to those of the white blood cell (WBC) count, neutrophil count, and percentage of neutrophils. Although the C-statistic of the DNI at time-0 was lower than those of Troponin-T (Tn-T) and N-terminal pro B-type natriuretic peptide (NT-proBNP) at admission, the DNI at time-I was not significantly inferior to those of creatine kinase-MB (CK-MB), Tn-T, and NT-proBNP at admission. The DNI at time-24 was better at predicting 30-day mortality than CK-MB or C-reactive protein (CRP) measured 24 h post-admission (Fig. 2B).

**Table 2:** Univariable Cox proportional hazard regression analysis for predictors of 30-day mortality.

Variables	Univariable cox proportional hazard regression	
	HR (95% CI)	*p*-value
Male sex (vs female)	0.511 (0.325–0.804)	0.004*
Body mass index (per 1 kg/m²)	0.954 (0.895–1.017)	0.147
Use of LMWH	0.530 (0.074–3.808)	0.528
Use of unfractionated heparin	0.137 (0.043–0.434)	<0.001*
GRACE score (per 1 point)	1.031 (1.025–1.036)	<0.001*
Age (per 1 year)	1.057 (1.037–1.076)	<0.001*
Heart rate (per 1 beat/min)	1.001 (0.993–1.010)	0.801
SBP (per 1 mmHg)	0.982 (0.978–0.986)	<0.001*
Creatinine (per 1 mg/dL)	1.183 (1.082–1.294)	<0.001*
Troponin-T (per 10^3 pg/mL)	1.274 (1.190–1.364)	<0.001*
Arrest on admission	8.657 (4.693–15.967)	<0.001*
**Killip class**		
I	Reference (1.000)	
II	1.061 (0.369–3.053)	0.913
III	7.145 (3.499–14.587)	<0.001*
IV	10.266 (5.309–19.853)	<0.001*
**Clinical measurements**		
LVEF (per 1%)	0.900 (0.885–0.915)	<0.001*
NT-proBNP (per 10^3 pg/mL)	1.042 (1.029–1.055)	<0.001*
Creatine kinase-MB (per 1 ng/mL)	1.004 (1.003–1.006)	<0.001*
White blood cell count (per 10^3/μL)	1.056 (1.008–1.107)	0.022*
Neutrophil ratio (per 1%)	1.009 (0.996–1.022)	0.198
hs-CRP (per 1 mg/L)	1.002 (0.999–1.005)	0.188
aPTT (per 1 sec)	1.005 (1.000–1.010)	0.065
Platelets (per 10^3/μL)	0.997 (0.994–1.000)	0.036*
Total cholesterol (per 1 mg/dL)	0.992 (0.987–0.997)	<0.001*
Triglyceride (per 1 mg/dL)	0.997 (0.993–1.001)	0.104
Glucose (per 1 mg/dL)	1.004 (1.003–1.006)	<0.001*
**Medical History**		
Hypertension	1.125 (0.734–1.723)	0.590
Diabetes mellitus	1.692 (1.094–2.618)	0.018*
COPD	1.167 (0.287–4.745)	0.829
Hyperlipidaemia	0.480 (0.195–1.186)	0.112
History of PCI	0.936 (0.484–1.810)	0.844
CAOD	1.623 (0.991–2.657)	0.054
Heart failure	1.464 (0.463–4.635)	0.516
Arrhythmia	0.471 (0.066–3.381)	0.454
Stroke	3.400 (1.846–6.261)	<0.001*
PAOD	1.768 (0.435–7.187)	0.426
Malignancy	2.079 (1.042–4.149)	0.038*
Chronic kidney disease	2.216 (1.111–4.421)	0.024*
Chronic liver disease	1.431 (0.199–10.276)	0.722
**Procedural characteristics**		
Door-to-balloon time (per 1 min)	1.006 (1.002–1.010)	<0.001*
Procedure time (per 1 min)	1.013 (1.007–1.020)	<0.001*
**Type of contrast medium**		
Iopamidol	Reference (1000)	
Iodixanol	0.921 (0.562–1.508)	0.743
Iopromide	4.710 (0.633–35.021)	0.13
Contrast volume (per 1 mL)	0.999 (0.996–1.002)	0.501
Multivessel disease	1.869 (1.150–3.036)	0.012*
LM artery involvement	2.915 (1.507–5.640)	0.002*
DNI Time-0 (per 1%)	1.165 (1.096–1.239)	<0.001*
DNI Time-I (per 1%)	1.118 (1.101–1.136)	<0.001*
DNI Time-24 (per 1%)	1.092 (1.079–1.106)	<0.001*

HR, hazard ratio; CI, confidence interval; LMWH, low molecular weight heparin; GRACE, Global Registry of Acute Coronary Events; SBP, systolic blood pressure; LVEF, left ventricular ejection fraction; NT-proBNP, N-terminal pro-brain natriuretic peptide; hs-CRP, high-sensitivity C-reactive protein; aPTT, activated partial thromboplastin time; COPD, chronic obstructive pulmonary disease; PCI, Percutaneous Coronary Intervention; CAOD, coronary artery occlusive disease; PAOD, peripheral arterial occlusive disease; LM, left main coronary artery; DNI, delta neutrophil index. *P < 0.05.

**Table 3:** Multivariable Cox proportional hazard regression analysis for predictors of 30-day mortality.

Variable	Multivariable cox proportional hazard regression analysis (30-day mortality)					
	HR (95% CI)	*p*-value	HR (95% CI)	*p*-value	HR (95% CI)	*p*-value
Male sex (vs female)	0.613 (0.360–1.044)	0.071	0.411 (0.228–0.739)	0.003*	0.648 (0.334–1.256)	0.199
Use of unfractionated heparin	0.079 (0.022–0.285)	<0.001*	90878.70 (0.000-.)	0.986	0.301 (0.054–1.677)	0.171
GRACE score (per 1 point)	1.028 (1.020–1.036)	<0.001*	1.021 (1.013–1.029)	<0.001*	1.016 (1.006–1.025)	0.001*
LVEF (per 1%)	0.923 (0.904–0.943)	<0.001*	0.936 (0.914–0.957)	<0.001*	0.933 (0.910–0.956)	<0.001*
NT-proBNP (per 10^3 pg/mL)	1.016 (0.944–1.039)	0.151	1.017 (0.994–1.041)	0.151	1.025 (1.001–1.051)	0.044*
Creatine kinase-MB (per 1 ng/mL)	1.002 (1.000–1.005)	0.062	1.002 (1.000–1.005)	0.067	1.004 (1.001–1.008)	0.014*
White blood cell count (per 10^3/μL)	1.012 (0.952–1.075)	0.71	1.004 (0.946–1.067)	0.889	1.004 (0.937–1.075)	0.912
Platelets (per 10^3/μL)	0.995 (0.992–0.999)	0.004*	0.997 (0.994–1.000)	0.053	0.998 (0.994–1.001)	0.178
Total cholesterol (per 1 mg/dL)	1.005 (1.000–1.010)	0.074	1.003 (0.997–1.008)	0.355	0.999 (0.992–1.006)	0.841
Glucose (per 1 mg/dL)	1.000 (0.998–1.003)	0.814	0.999 (0.996–1.002)	0.573	1.000 (0.997–1.003)	0.912
**Medical History**						
Diabetes mellitus	0.864 (0.472–1.583)	0.637	1.165 (0.595–2.281)	0.657	1.021 (0.497–2.098)	0.955
Stroke	1.661 (0.785–3.518)	0.185	2.113 (0.946–4.720)	0.068	3.114 (1.329–7.299)	0.009*
Malignancy	3.258 (1.371–7.744)	0.008*	2.415 (1.003–5.818)	0.049*	3.888 (1.621–9.328)	0.002*
Chronic kidney disease	1.030 (0.424–2.503)	0.949	1.034 (0.416–2.566)	0.943	0.862 (0.301–2.468)	0.783
Door-to-balloon time (per 1 min)	1.011 (1.004–1.018)	0.003*	1.010 (1.003–1.017)	0.006*	1.009 (1.001–1.017)	0.02*
Procedure time (per 1 min)	1.009 (0.998–1.020)	0.121	1.008 (0.996–1.021)	0.211	1.004 (0.990–1.019)	0.558
Multivessel disease	1.064 (0.599–1.892)	0.832	0.900 (0.482–1.678)	0.74	1.139 (0.578–2.243)	0.706
LM artery involvement	2.330 (1.103–4.920)	0.027*	3.028 (1.409–6.506)	0.005*	3.068 (1.404–6.702)	0.005*
DNI Time-0 (per 1%)	1.019 (0.916–1.133)	0.729				
DNI Time-I (per 1%)			1.076 (1.046–1.108)	<0.001*		
DNI Time-24 (per 1%)					1.066 (1.045–1.086)	<0.001*

HR, hazard ratio; CI, confidence interval; GRACE, Global Registry of Acute Coronary Events; LVEF, left ventricular ejection fraction; NT-proBNP, N-terminal pro-brain natriuretic peptide; LM, left main coronary artery; DNI, delta neutrophil index. *P < 0.05.

The integrated discrimination improvement (IDI) and continuous net reclassification improvement (NRI) are proposed indicators to identify improvement of reclassification in a nested model, thus showing how the predictive power is improved when the DNI is added to traditional risk factors. If this value is greater than ‘0’, the DNI can be determined as a factor that can improve the predictive power when added to existing prediction models (Fig. 2A). The addition of the DNI yielded a significantly positive IDI for the DNI values at times I and 24. The continuous NRI was also positive; it was significant for the DNI at time-I and showed an increased trend for the DNI at time-24.

To estimate the optimal cut-off values based on time-to-event data, Kaplan-Meier curves of 30-day mortality were generated based on the DNI values at times 0, I, and 24. These DNI values were also found to be independent predictors of clinical outcomes within 30 days post-pPCI. Specifically, an increased risk of 30-day mortality was observed among patients with an increased DNI at each time point after ED admission.

**Figure 3:**
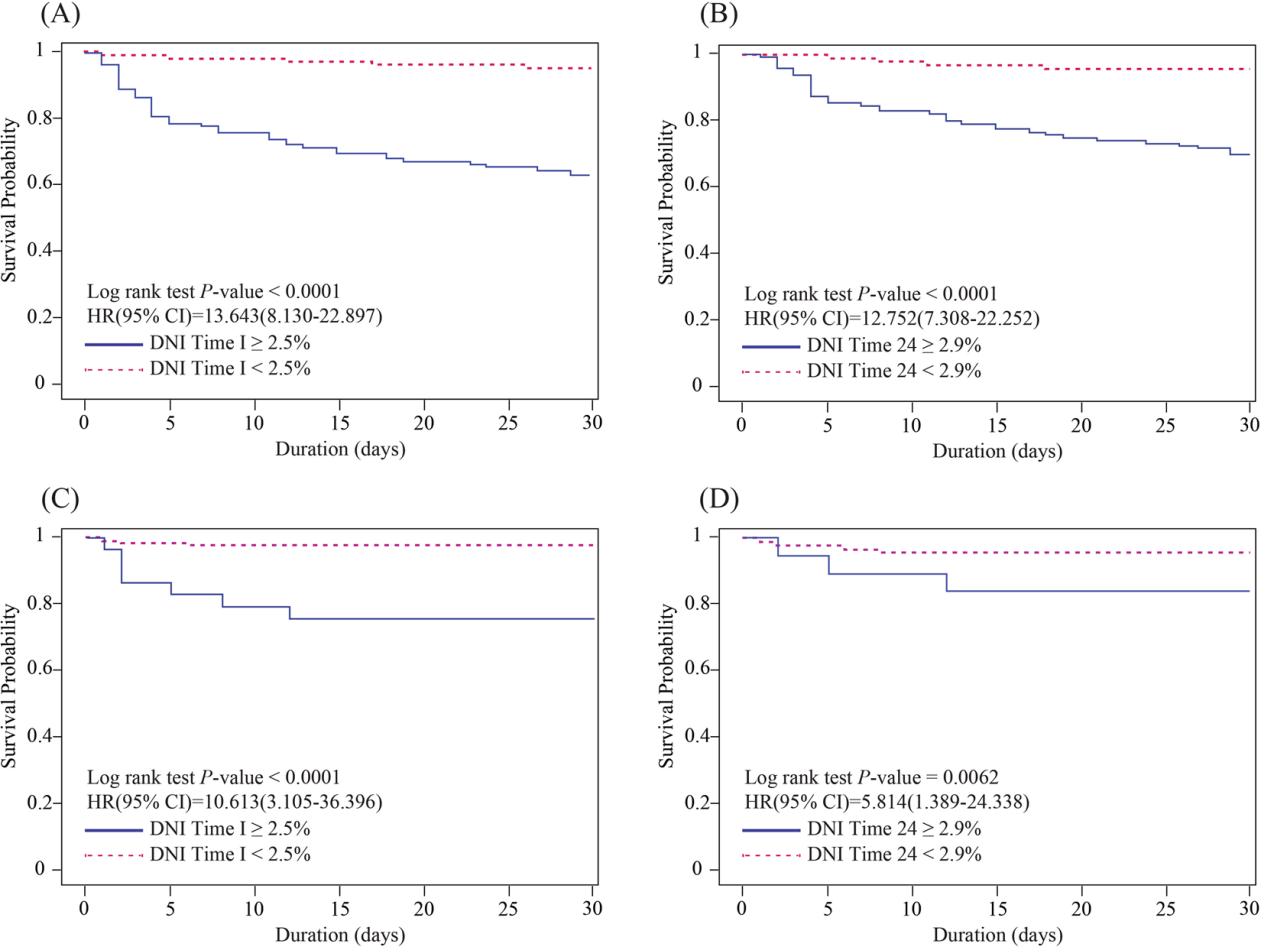
The delta neutrophil index (DNI) as a predictor of 30-day mortality. Higher DNI values within 2 h post-primary percutaneous coronary intervention (pPCI) (**A**) and 24 hours after emergency department admission (**B**) were significantly associated with increased 30-day mortality risk among patients with ST segment elevation myocardial infarction (STEMI) who underwent pPCI. When the same cut-offs were applied to the validation cohort, external validation demonstrated that higher DNI values within 2 h post-pPCI (**C**) and 24 hours after emergency department admission (**D**) remained significantly associated with increased risks of 30-day mortality. HR, hazard ratio; CI, confidence interval.

The log-rank test indicated that the optimal DNI cut-off values for 30-day mortality predictions were 2.5% at time-I (p < 0.001) and 2.9% at time-24 (p < 0.001). Further analysis of these cut-off values using the Contal and O’Quigley technique indicated that DNI values >2.5% at time-I (hazard ratio [HR], 13.643; 95% confidence interval [CI]: 8.13–22.897; p < 0.001) and >2.9% at time-24 (HR, 12.752; 95% CI: 7.308–22.252; p < 0.001) were associated with increased risks of 30-day mortality among patients with STEMI who underwent pPCI (Fig. 3A and B). When these cut-offs were applied to the validation cohort, DNI values >2.5% at time-I (HR, 10.616; 95% CI: 3.105–32.296; p < 0.001) and >2.9% at time-24 (HR, 5.814; 95% CI: 1.389–24.338; p = 0.016) remained significantly associated with increased risks of 30-day mortality (Supplement 4, Fig. 3C and D).

## Discussion

In AMI, profound systemic inflammation, caused by dysregulation of the immune system, is associated with increased inflammatory mediators and activation of peripheral leukocytes and neutrophils or neutrophil subtypes^6^. An intense inflammatory response is activated in the early stage of cardiac ischaemic injury; this contributes significantly to ventricular remodelling after AMI^3^.

Neutrophils are critical cells in innate immunity. They mediate tissue damage after ischaemia-reperfusion injury^6^. Tamhane *et al*. demonstrated that neutrophilia >65% in patients with STEMI reflected worse angiographic outcomes, large infarct size, and increased risk of short-term mortality^16^. Activated neutrophils damage the myocardium, leading to microvascular obstruction^17^. In fact, neutrophil plugging is the most critical cause of post-PCI major adverse cardiac events^17,18^. In addition, immature granulocytes enter the circulation during infection, stress, or systemic inflammation; the Systemic Inflammatory Response Syndrome criteria include an increase in the number of immature granulocytes in circulation^11,19^. Our study also demonstrated that the DNI value within 2 h after reperfusion (time-I) was superior to the WBC, neutrophil count, and percentage of neutrophils for predicting short-term mortality in patients with STEMI.

Many studies have attempted to propose and validate risk stratification systems for the identification of patients at high risk of death or with a critical prognosis^5,20^. Among several scoring systems, the Global Registry of Acute Coronary Events (GRACE) risk score, which includes two biomarkers (serum creatinine and troponin), is widely accepted to estimate prognosis^20^. Clinically, this risk score is able to differentiate critical patients at high risk of mortality and to predict the mortality rate in patients with STEMI in the ED setting^20,21^. However, the GRACE risk score is excessively complicated with respect to predicting early severity, and requires serial or multiple measurements to determine the severity. Serum biomarkers, such as NT-proBNP and high-sensitivity CRP (hs-CRP), are widely used to identify ischaemia-reperfusion injury and prognosis after reperfusion in patients with STEMI, assisting in the estimation of infarct size, microvascular obstruction, and left-ventricular remodelling, and in the stratification of risk in patients with AMI^22,23^. Both the peak CK-MB and peak Tn-I levels have been shown to be independently associated with in-hospital mortality^24^. However, the cost effectiveness of risk prediction must be considered in the requirement for serial measurements of cardiac-specific markers in the clinical setting^5^. The clinical utility of a biomarker for risk prediction depends on its practicability, ease, cost, and reproducibility of the measurement, as well as on the ability to add it to existing biomarkers to improve the predictability^25^.

Nahm *et al*. demonstrated that the DNI was strongly correlated with the manual immature granulocyte counts and that the use of an automated blood cell analyser for calculating the DNI can overcome the limitations of delay and poor accuracy of manual counting of immature granulocytes^11,15^. The present study showed that changes in the DNI values over time were associated with poor clinical outcomes in patients with STEMI, including 30-day mortality. Similarly, Yune *et al*. reported that a DNI >8.4% on admission (HR, 3.227) and DNI >10.5% on day 1 (HR, 3.292) were associated with increased 30-day mortality in patients surviving out-of-hospital cardiac arrest^11^. Thus, the authors concluded that an increased DNI reflects increased severity of systemic and sterile inflammation^11^.

In terms of sepsis, a previous study by Park *et al*. revealed that a DNI >6.5% was a good diagnostic marker of severe sepsis and septic shock within the first 24 h after intensive care unit admission^10^. Previous studies have proposed potential mechanisms to explain this rapid and early release of immature granulocytes. In cases of sterile inflammation, such as AMI or post-resuscitation after out-of-hospital cardiac arrest, the mechanism for increasing immature granulocytes is likely similar to that in sepsis. For example, the rapid expansion of circulating neutrophils to compensate for the loss of active neutrophils due to the massive consumption and destruction of mature cells in severe inflammation is one likely mechanism^19,26–29^. Further, in myocardial reperfusion injury, reperfusion induces endothelial dysfunction, which results in vasoconstriction during the first few minutes, while the increased leukocyte adhesion and influx result in impaired blood flow^30^. Death of cardiomyocytes can be directly induced by these leukocytes^30^. In addition, neutrophil paralysis—known as dysregulated neutrophil function—attenuates tissue damage in severe sterile inflammation as a result of impaired migration of neutrophils to the injured site and neutrophil sequestration in remote organs^26,27^. Sauneuf *et al*. suggested bone marrow exhaustion as a further mechanism by which severe ischaemia-induced inflammation could lead to a transient failure of the regulation of neutrophil release during ischaemia and following resuscitation^28,31^. In particular, hemodynamic instability or persistently severe inflammation, according to an increase in the severity of STEMI, may affect critical regulatory mechanisms for neutrophil release from the bone marrow. This, the DNI after reperfusion may be a promising biomarker for predicting severity and mortality in the early stages of STEMI.

In this study of patients with STEMI who underwent pPCI, we found that the DNI was a significant independent predictor of 30-day mortality. Specifically, we found that DNI values >2.5% immediately (within 2 h) after pPCI and >2.9% at 24 h post-admission (times I and 24, respectively) could significantly predict 30-day mortality in this group of patients. We also assessed PCI-related variations of the DNI in patients admitted with non-STEMI or unstable angina who underwent elective PCI. The mean DNI values immediately after pPCI were more rapidly increased in the non-survival group, who died within 30 days, than in the survival group at admission. Hence, we propose the use of the DNI, which can be determined rapidly, easily, and inexpensively, to assess severity in such patients.

Our study moreover revealed that the DNI within 2 h post-reperfusion had similar predictability as CK-MB, Tn-T, and NT-proBNP for 30-day mortality. In addition, the DNI value 24 h post-admission was superior to CK-MB and hs-CRP for predicting 30-day mortality. The DNI has the added benefit of being automatically analysed with the CBC, which is routinely and immediately performed in critically ill patients, without additional time or cost, unlike hs-CRP, CK-MB, and Tn-T^11^. Thus, we propose that the DNI, by reflecting systemic inflammation, may be a promising marker for the assessment of severity in patients with STEMI after pPCI. In the future, prospective multi-centre studies with a larger number of patients will be needed to validate our findings.

This study has several limitations, including its retrospective design, and the inclusion of a patient cohort derived from a single, tertiary, academic hospital. Therefore, it was difficult to control for confounding factors, thus increasing the risk of selection bias. However, we used a critical pathway that was prospectively performed with a standardized and predetermined protocol. Second, we could not accurately assess the long-term clinical outcomes. Third, heparin has been shown to inhibit neutrophil activation and induce the aggregation and apoptosis of neutrophils^32,33^. In the present study, we could not compare the clinical effects of heparin on the DNI over time because all patients received unfractionated or low molecular weight heparin before the PCI procedure. Further molecular studies are needed to validate the effects of heparin on the DNI over time, considering the effects of heparin on the activation, aggregation, and apoptosis of neutrophils. Fourth, Pencina *et al*. suggested that NRI values of <0.2, 0.2–0.6, and >0.6, should be considered weak, intermediate, and strong, respectively. In this study, the NRI value when adding the DNI to the null model was approximately 0.05–0.22, showing only weak reclassification ability according to these criteria. However, it should be noted that Harrell’s C-index (95% CI) of the null model (0.943 [0.923–0.962]) showed very strong prognostic power, because the null model comprises a combination of strong predictors. Thus, considering the great advantages of the DNI, the DNI may still be clinically valuable in patients with STEMI despite the relatively weak NRI in the present study. Finally, despite using a prospective registry, serial measurements for indicators of severity of STEMI (such as CK-MB, Tn-T, hs-CRP, and proinflammatory cytokines) are not mandatory in our FIRST protocol. Therefore, we were unable to serially evaluate all indicators of severity of STEMI at the same time points as the DNI values were measured. Further studies are required to validate and compare the usefulness of these indicators of severity as prognostic markers in patients with STEMI.

In summary, we found that an increased DNI level, which reflects the proportion of circulating immature granulocytes in the blood, is an independent predictor of 30-day mortality and poor clinical outcomes in patients with acute STEMI post-pPCI. The DNI can be obtained without additional costs or time burdens, and can be measured rapidly and simply after ED admission, indicating its clinical usefulness. Patients with a high DNI level after PCI should be cautiously monitored to implement the appropriate treatment strategies.

## Methods

### Study population and the Fast Interrogation Rule for ST-elevation Myocardial Infarction (FIRST) protocol

This retrospective, observational cohort study was conducted between January 1, 2011 and June 30, 2017 at Yonsei University College of Medicine, Severance Hospital, a single tertiary academic hospital that attends to 85,000 patients in the emergency department (ED) annually. The study was reviewed and approved by the institutional review board of Yonsei University Health System (3–2015–0140).

In 2007, a multidisciplinary critical pathway based on a computerized provider order entry (CPOE) system, known as FIRST, was implemented in our institution. Our critical pathway for STEMI management was designed to reduce unnecessary in-hospital time delays through a CPOE-based alert system, short message service, and simple standing orders through the activation stage. The present study included consecutive patients who were prospectively integrated into the FIRST critical pathway program, with those admitted with STEMI and who underwent pPCI between January 1, 2011 and June 30, 2017 being analysed. Figure 1 and Supplement 5 summarize the inclusion and exclusion criteria.

Upon arrival of a patient to the ED, the physicians, nurses, and emergency medical technicians in the triage area identified candidates for the FIRST program as soon as possible according to pre-determined protocols. Simultaneously, a 12-lead ECG was performed in the triage area^34^. When a patient had at least one predetermined ECG warning criterion for STEMI on ED arrival, within 12-h of the onset of symptoms, the triage ED physician activated the FIRST program by selecting the activation icon on the order entry window (Supplement 5)^34^. Once activated, the on-call cardiologist was consulted. The on-call cardiologist immediately assessed the patient and applied standard treatment in accordance with the guideline of the American College of Cardiology Foundation/American Heart Association. Coronary angiography and PCI were conducted using standard protocols and guidelines.

### Data collection

We examined data related to the patients’ demographics, laboratory test results (including cardiac enzymes), volume of contrast medium, PCI findings, procedure time, left ventricular ejection fraction, and presence of multivessel disease based on a predetermined protocol. We also evaluated the Killip classification, GRACE score, and the door-to-balloon time interval. Venous blood was collected in ethylenediaminetetraacetic-containing vacutainers on presentation to the ED and at multiple time points (within 2 h of reperfusion and 24 h after ED admission) for measurements of the DNI, using the same type of haematology analyser used for the analysis of the CBC.

### DNI and other blood sample measurements

The CBC, comprising the DNI, WBC count, haemoglobin level, and platelet count, was analysed by an automated blood cell analyser (ADVIA 2120; Siemens, Forchheim, Germany). This analyser comprises two independent WBC analysis methods using flow cytometric principles. First, the optical system based on the cytochemical myeloperoxidase tungsten-halogen channel measures and differentiates granulocytes, lymphocytes, and monocytes based on size and myeloperoxidase content staining intensity^10,15^. Second, the optical system, using the lobularity/nuclear density channel laser-diode, calculates and classifies the cell types with respect to their lobularity/nuclear density and size^10,15^. The DNI is then calculated by subtracting the fraction of mature polymorphonuclear neutrophils from the sum of the myeloperoxidase-reactive cells, detecting circulating immature granulocytes as the leukocyte subfraction (Supplement 6)^10,15^. Other laboratory tests conducted at the time of ED admission included determination of blood urea nitrogen, creatinine, alanine transaminase, hs-CRP, CK, CK-MB, Tn-T, NT-proBNP, and albumin levels, assessed using an automated chemistry analyser.

### Clinical outcomes

The primary clinical outcome was 30-day mortality. In addition, we analysed other clinically important outcomes, including the development of heart failure, defined as new episodes of congestive heart failure on the basis of clinical findings consistent with this diagnosis documented in the medical records, in association with echocardiographic evidence of contractile dysfunction (ejection fraction <40%)^35,36^.

### Statistical analysis

Demographic and clinical data are presented as the median (interquartile range), mean ± standard deviation, and percentage or frequency, as appropriate. Continuous variables were compared using a two-sample *t*-test or the Mann–Whitney U-test. Categorical variables were compared using the χ^2^ test or Fisher’s exact test. We estimated significant differences between groups over time using a linear mixed model and a repeated measures covariance pattern with unstructured covariance. Two fixed effects were included to address the clinical effect (level: survival and death) and time effect (time: DNI performed on admission, immediately after pPCI, and 24 h and 48 h after ED admission). Differences in the clinical effect over time were analysed according to the clinical effect × time. In addition, we also analysed differences in the development of heart failure over time using the same approach.

Next, we identified promising independent predictive factors of 30-day mortality by considering time-to-event data in patients with STEMI undergoing pPCI using a multivariable Cox proportional hazard regression model that integrated all major covariates (variables with p < 0.05) identified in our univariable analyses. The results are expressed as HRs and 95% CIs.

Moreover, univariable analyses were conducted to evaluate the relationships among demographic characteristics and clinical data. To highlight independent indicators of prognosis, we determined the independent prognostic factors of new-onset heart failure among patients with STEMI using multivariable logistic regression analysis, integrating major covariates (variables with a *p* value < 0.05) indicated from our univariable analysis. The results are expressed as odds ratios and 95% CIs. To identify the relationship between the DNI and the left ventricular ejection fraction, we performed Pearson correlation analysis.

To investigate the additional predictive power of the DNI at each time point, we calculated Harrell’s C-index for each Cox regression model^37–40^. To calculate the 95% CIs and p-values for the C index and the differences between models, we used a standard bootstrap method with resampling 1,000 times^37^. We also assessed the continuous NRI and IDI at the median follow-up time (5 days) to assess the improvement in performance of the survival model with the addition of the DNI^37,41,42^. The IDI, NRI, and C-statistic were calculated by bootstrapping (1,000 repetitions). We compared the C index to assess at which time point the DNI provided the better prognostic value. Kaplan-Meier survival curves were created using 30-day mortality data, and the groups were compared using the log-rank test. Although previous studies estimated the cut-off values based only on events, we estimated the optimal cut-off values for the dichotomization of the clinical outcome variable based on time-to-event data using the technique devised by Contal and O’Quigley. The optimal cut-off points were selected by maximizing the HR. To identify the validity of the cut-off points, we performed external validation using a validation cohort of Yonsei University College of Medicine affiliated Gangnam Severance Hospital.

All statistical analyses were performed using SAS, version 9.2 (SAS Institute Inc., Cary, NC), R software, version 3.2.5 for Windows (the R foundation for statistical computing, Vienna, Austria), and MedCalc, version 12.7.0 (MedCalc Software, Ostend, Belgium). For all analyses, a p-value < 0.05 was considered significant.

### Ethics statement

The study was reviewed and approved by the institutional review board of Yonsei University Health System (3-2015-0140). The requirement for written informed consent from the patients was waived because of the retrospective study design, the fact that the tests performed formed a part of the current standard of care in our ED, and as the patient data were anonymous. Our study was conducted according to the ethical standards laid down in the 1964 Declaration of Helsinki and its later amendments.

### Data availability statement

The datasets generated and/or analysed during the current study are available from the corresponding author on reasonable request.

## Electronic supplementary material

Supplementary Information
