# Supplementary material for: Usefulness of the delta neutrophil index to predict 30-day mortality in patients with ST segment elevation myocardial infarction
Source: Sci Rep. 2017 Nov 16;7:15718. doi: 10.1038/s41598-017-15878-5 (PMC5691079; doi:10.1038/s41598-017-15878-5)
Supplement: Supplementary file 1 — Supplementary Information [file 41598_2017_15878_MOESM1_ESM.pdf]

Supplementary Information

Usefulness of the delta neutrophil index to predict 30-day mortality in patients with ST segment elevation myocardial infarction

Taeyoung Kong, MD[1], Tae Hoon Kim, MD[2], Yoo Seok Park, MD, PhD[1], Sung Phil Chung, MD, PhD[1], Hye Sun Lee PhD[3], Jung Hwa Hong, MS[4], Jong Wook Lee, MD[5,6], Je Sung You, MD, PhD[1*], Incheol Park, MD, PhD[1]

1. Department of Emergency Medicine, Yonsei University College of Medicine, Seoul, Republic of Korea

2. Division of Cardiology, Department of Internal Medicine, Yonsei University College of Medicine, Seoul, Republic of Korea

3. Department of Research Affairs, Biostatistics Collaboration Unit, Yonsei University College of Medicine, Seoul, Republic of Korea

4. Department of Health Insurance Research, National Health Insurance Service Ilsan Hospital, Gayang, Republic of Korea

5. Department of Laboratory Medicine, Konyan University Hospital, Daejeon, Republic of Korea

6. Research Institute of Bacterial Resistance, Yonsei University College of Medicine, Seoul, Korea

Correspondence author: Je Sung You, MD, PhD*

Department of Emergency Medicine, Yonsei University College of Medicine

211 Eonju-Ro, Gangnam-Gu, Seoul 135-720, Republic of Korea

E-mail: youjsmd@yuhs.ac

TEL: 82-2-2019-3030, FAX: 82-2-2019-4820

Variables	Development of new-onset heart failure				
	Yes (N=218)	No (n=624)	*p*-value	OR (95% CI)	*p*-value
**Male sex (vs female)**	172(78.90)	495(79.33)	0.893	0.974(0.667-1.423)	0.893
**Body mass index (per 1 kg/m²)**	23.79±3.60	24.34±3.39	0.044*	0.954(0.911-0.999)	0.045*
**Use of LMWH**	6(2.75)	12(1.92)	0.428	1.444(0.535-3.894)	0.468
**Use of unfractionated heparin**	217(99.54)	619(99.20)	0.999	1.749(0.204-15.038)	0.61
**GRACE score (per 1 point)**	195.64±45.31	160.64±43.79	<0.001*	1.017(1.013-1.021)	<0.001*
**Age (per 1 years)**	65.78±13.33	62.55±13.12	0.002*	1.019(1.007-1.031)	0.002*
**Heart rate (per 1 beat/min)**	88.74±34.19	78.38±22.51	<0.001*	1.016(1.009-1.022)	<0.001*
**SBP (per 1 mmHg)**	115.36±43.06	130.48±36.30	<0.001*	0.990(0.986-0.994)	<0.001*
**Creatinine (per 1 mg/dL)**	1.42±1.44	1.18±1.13	0.025*	1.150(1.025-1.289)	0.017*
**Troponin-T (per 10^3 pg/mL)**	1259.65±2305.23	467.74±1289.43	<0.001*	1.286(1.174-1.409)	<0.001*
**Arrest on admission**	12(5.50)	10(1.60)	0.002*	3.577(1.523-8.401)	0.003*
**Killip class**			<0.001*		
I	59(27.06)	321(51.44)		Reference (1)	
II	29(13.30)	135(21.63)		1.169(0.718-1.904)	0.531
III	49(22.48)	76(12.18)		3.508(2.228-5.523)	<0.001*
IV	81(37.16)	92(14.74)		4.790(3.186-7.202)	<0.001*
**Clinical measurements**					
**LVEF (per 1%)**	30.49±8.29	50.44±8.79	<0.001*	0.649(0.603-0.697)	<0.001*
**NT-proBNP (per 10^3 pg/mL)**	6020.7±10929.1	2101.1±6596.3	<0.001*	1.055(1.034-1.076)	<0.001*
**Creatine kinase-MB (per 1 ng/mL)**	49.01±98.04	21.37±50.92	<0.001*	1.005(1.003-1.008)	<0.001*
**White blood cell count (per 10^3/μL)**	12.57±4.19	11.09±3.90	<0.001*	1.091(1.051-1.133)	<0.001*
**Neutrophil ratio (per 1%)**	67.52±17.93	64.17±16.66	0.013*	1.012(1.003-1.021)	0.013*
**hs-CRP (per 1 mg/L)**	38.44±66.58	20.40±48.51	<0.001*	1.005(1.003-1.008)	<0.001*
**aPTT (per 1 s)**	35.46±26.86	35.70±29.46	0.917	1.000(0.994-1.005)	0.918
**Platelets (per 10^3/μL)**	247.18±102.91	243.30±73.13	0.608	1.001(0.999-1.002)	0.547
**Total cholesterol (per 1 mg/dL)**	176.96±44.34	189.37±48.28	<0.001*	0.994(0.991-0.998)	0.001*
**Triglyceride (per 1 mg/dL)**	100.10±54.97	120.76±114.51	<0.001*	0.997(0.994-0.999)	0.009*
**Glucose (per 1 mg/dL)**	229.84±115.55	184.70±82.38	<0.001*	1.005(1.003-1.006)	<0.001*
**Medical History**					
**Hypertension**	106(48.62)	328(52.56)	0.316	0.854(0.627-1.163)	0.317
**Diabetes mellitus**	84(38.53)	148(23.76)	<0.001*	2.012(1.447-2.797)	<.0001*
**COPD**	6(2.75)	11(1.76)	0.403	1.578(0.576-4.318)	0.375
**Hyperlipidaemia**	14(6.42)	80(12.82)	0.01*	0.467(0.259-0.842)	0.011*
**History of PCI**	24(11.01)	82(13.14)	0.414	0.818(0.504-1.326)	0.415
**CAOD**	38(17.43)	107(17.15)	0.924	1.020(0.679-1.533)	0.924
**Heart failure**	1(0.46)	20(3.21)	0.025*	0.139(0.019-1.043)	0.055
**Arrhythmia**	3(1.38)	17(2.72)	0.260	0.498(0.145-1.717)	0.27
**Stroke**	14(6.42)	29(4.65)	0.306	1.408(0.730-2.717)	0.307
**PAOD**	1(0.46)	10(1.60)	0.306	0.283(0.036-2.224)	0.23
**Malignancy**	12(5.50)	34(5.45)	0.975	1.011(0.514-1.989)	0.975
**Chronic kidney disease**	13(5.96)	31(4.97)	0.570	1.213(0.623-2.363)	0.57
**Chronic liver disease**	0(0.00)	7(1.12)	0.199	0.000(0.000-1)	0.98
**Procedural characteristics**					
**Door-to-balloon time (per 1 min)**	69.12±36.61	63.04±30.88	0.029*	1.005(1.001-1.010)	0.023*
**Procedure time (per 1 min)**	43.22±19.88	39.55±20.56	0.023*	1.008(1.001-1.015)	0.025*
**Type of contrast medium**			0.182		
**Iopamidol**	59(27.06)	139(22.28)		Reference (1)	
**Iodixanol**	158(72.48)	484(77.56)		0.769(0.540-1.095)	0.146
**Iopromide**	1(0.46)	1(0.16)		2.353(0.145-38.243)	0.548
**Contrast volume (per 1 mL)**	196.51±90.85	191.43±70.13	0.453	1.001(0.999-1.003)	0.396
**Multivessel disease**	154(70.64)	361(57.85)	<0.001*	1.753(1.258-2.443)	<0.001*
**LM artery involvement**	20(9.17)	18(2.88)	<0.001*	3.401(1.763-6.558)	<0.001*
**DNI Time-0 (per 1%)**	1.06±2.32	0.68±1.56	0.025*	1.110(1.026-1.201)	0.01*
**DNI Time-I (per 1%)**	5.54±8.38	1.10±2.83	<0.001*	1.278(1.200-1.362)	<0.001*
**DNI Time-24 (per 1%)**	6.44±12.13	1.14±2.42	<0.001*	1.225(1.151-1.304)	<0.001*

Supplement 1. Clinical characteristics and univariable logistic regression analysis for the predictors of the development of new-onset heart failure

OR, odds ratio; CI, confidence interval; LMWH, low molecular weight heparin; GRACE, Global Registry of Acute Coronary Events; SBP, systolic blood pressure; LVEF, left ventricular ejection fraction; NT- proBNP, N-terminal pro-brain natriuretic peptide; hs-CRP, high-sensitivity C-reactive protein; aPTT, activated partial thromboplastin time; COPD, chronic obstructive pulmonary disease; PCI, Percutaneous Coronary Intervention; CAOD, coronary artery occlusive disease; PAOD, peripheral arterial occlusive disease; LM, left main coronary artery; DNI, delta neutrophil index. Data are expressed as the mean ± standard deviation or number (percentage). *$P<0.05$.

Variable	Multivariable logistic regression analysis (Development of new-onset heart failure)					
	OR (95% CI)	*p*-value	OR (95% CI)	*p*-value	OR (95% CI)	*p*-value
**Body mass index (per 1 kg/m²)**	0.991(0.938-1.046)	0.734	1.005(0.948-1.066)	0.863	0.982(0.927-1.040)	0.531
**GRACE score (per 1 point)**	1.012(1.007-1.017)	<0.001*	1.011(1.006-1.016)	<0.001*	1.007(1.002-1.013)	0.007*
**NT-pro BNP (per 10^3 pg/mL)**	1.017(0.994-1.041)	0.154	1.020(0.995-1.045)	0.115	1.018(0.993-1.044)	0.149
**Creatine kinase-MB (per 1 ng/mL)**	1.004(1.002-1.007)	0.001*	1.005(1.002-1.008)	0.001*	1.005(1.002-1.008)	0.002*
**White blood cell count (per 10^3/μL)**	1.079(1.026-1.136)	0.003*	1.057(1.003-1.114)	0.039*	1.061(1.007-1.118)	0.026*
**Neutrophil ratio (per 1%)**	1.003(0.990-1.016)	0.663	1.004(0.990-1.018)	0.556	1.003(0.990-1.017)	0.637
**hs-CRP (per 1 mg/L)**	1.002(0.998-1.005)	0.326	1.002(0.998-1.005)	0.391	1.001(0.998-1.005)	0.399
**Total cholesterol (per 1 mg/dL)**	1.000(0.996-1.005)	0.951	1.002(0.997-1.007)	0.393	1.001(0.996-1.006)	0.653
**Glucose (per 1 mg/dL)**	1.002(1.000-1.004)	0.122	1.000(0.998-1.003)	0.753	1.001(0.998-1.003)	0.514
**Medical History**						
**Diabetes mellitus**	1.386(0.886-2.167)	0.153	1.809(1.122-2.915)	0.015*	1.667(1.034-2.688)	0.036*
**Hyperlipidemia**	0.414(0.212-0.810)	0.01*	0.454(0.224-0.919)	0.028*	0.470(0.235-0.940)	0.033*
**Door-to-balloon time (per 1 min)**	1.003(0.998-1.009)	0.249	1.004(0.999-1.010)	0.129	1.003(0.997-1.009)	0.32
**Procedure time (per 1 min)**	0.999(0.991-1.008)	0.892	1.000(0.990-1.010)	0.937	0.997(0.987-1.006)	0.498
**Multivessel disease**	1.473(0.992-2.187)	0.055	1.486(0.972-2.273)	0.067	1.432(0.940-2.183)	0.095
**LM artery involvement**	3.135(1.503-6.543)	0.002*	3.430(1.612-7.299)	0.001*	2.900(1.343-6.262)	0.007*
**DNI Time-0 (per 1%)**	0.936(0.832-1.053)	0.274				
**DNI Time-I (per 1%)**			1.137(1.070-1.208)	<0.001*		
**DNI Time-24 (per 1%)**					1.136(1.074-1.202)	<0.001*

Supplement 2. Multivariable logistic regression analysis for the predictors of development for new-onset heart failure

OR, odds ratio; CI, confidence interval; GRACE, Global Registry of Acute Coronary Events; NT-proBNP, N-terminal pro-brain natriuretic peptide; hs-CRP, high-sensitivity C-reactive protein; LM, left main coronary artery; DNI, delta neutrophil index. *P<0.05.

Variables	Harrell's C-index (95% CI)	Comparison model	Difference of Harrell's C-index (95% CI)	*P*-value
**DNI Time-0**	**0.562 (0.5-0.627)**			
Neutrophil ratio Time-0	0.556 (0.481-0.625)	(DNI Time-0) - (Neutrophil ratio Time-0)	0.007 (-0.067-0.083)	0.854
Neutrophil count Time-0	0.559 (0.5-0.622)	(DNI Time-0) - (Neutrophil count Time-0)	0.003 (-0.068-0.066)	0.932
WBC count Time-0	0.562 (0.503-0.626)	(DNI Time-0) - (WBC count Time-0)	0 (-0.077-0.076)	>0.999
**DNI Time-I**	**0.837 (0.781-0.888)**			
Neutrophil ratio Time-I	0.554 (0.422-0.652)	(DNI Time-I) - (Neutrophil ratio Time-I)	0.283 (0.163-0.428)	<0.001*
Neutrophil count Time-I	0.633 (0.564-0.703)	(DNI Time-I) - (Neutrophil count Time-I)	0.204 (0.125-0.281)	<0.001*
WBC count Time-I	0.648 (0.581-0.714)	(DNI Time-I) - (WBC count Time-I)	0.189 (0.115-0.262)	<0.001*
**DNI Time-24**	**0.845 (0.784-0.898)**			
Neutrophil ratio Time-24	0.762 (0.695-0.826)	(DNI Time-24) - (Neutrophil ratio Time-24)	0.083 (0.008-0.158)	0.025*
Neutrophil count Time-24	0.632 (0.54-0.713)	(DNI Time-24) - (Neutrophil count Time-24)	0.213 (0.112-0.317)	<0.001*
WBC count Time-24	0.582 (0.503-0.667)	(DNI Time-24) - (WBC count Time-24)	0.263 (0.16-0.369)	<0.001*
**DNI Time-I**	**0.837 (0.781-0.888)**			
CK-MB Time-0	0.629 (0.568-0.69)	(DNI Time-I) - (CK-MB Time-0)	0.208 (0.127-0.282)	<0.001*
Troponin T Time-0	0.682 (0.621-0.739)	(DNI Time-I) - (Troponin Time-0)	0.156 (0.077-0.234)	<0.001*
NT-pro BNP Time-0	0.725 (0.669-0.78)	(DNI Time-I) - (NT pro BNP Time-0)	0.113 (0.035-0.191)	0.006*
Hs-CRP Time-0	0.551 (0.475-0.625)	(DNI Time-I) - (hs C-reactive protein Time-0)	0.286 (0.195-0.375)	<0.001*
**DNI Time-24**	**0.845 (0.784-0.898)**			
CK-MB Peak value	0.552 (0.484-0.622)	(DNI Time-24) - (CK-MB Peak value)	0.293 (0.215-0.379)	<0.001*
Troponin T Peak value	0.695 (0.635-0.75)	(DNI Time-24) - (Troponin T Peak value)	0.151 (0.057-0.239)	0.001*
LVEF	0.839 (0.791-0.879)	(DNI Time-24) - (LVEF)	0.007 (-0.06-0.075)	0.837

Supplement 3. Comparison of biomarkers for the prediction of 30-day mortality using Harrell's C-index.

CI, confidence interval; DNI, delta neutrophil index; WBC, white blood cell; CK-MB, creatine kinase-MB; NT-proBNP, N-terminal pro-brain natriuretic peptide; hs-CRP, high-sensitivity C-reactive protein; LVEF, left ventricular ejection fraction. *P<0.05.

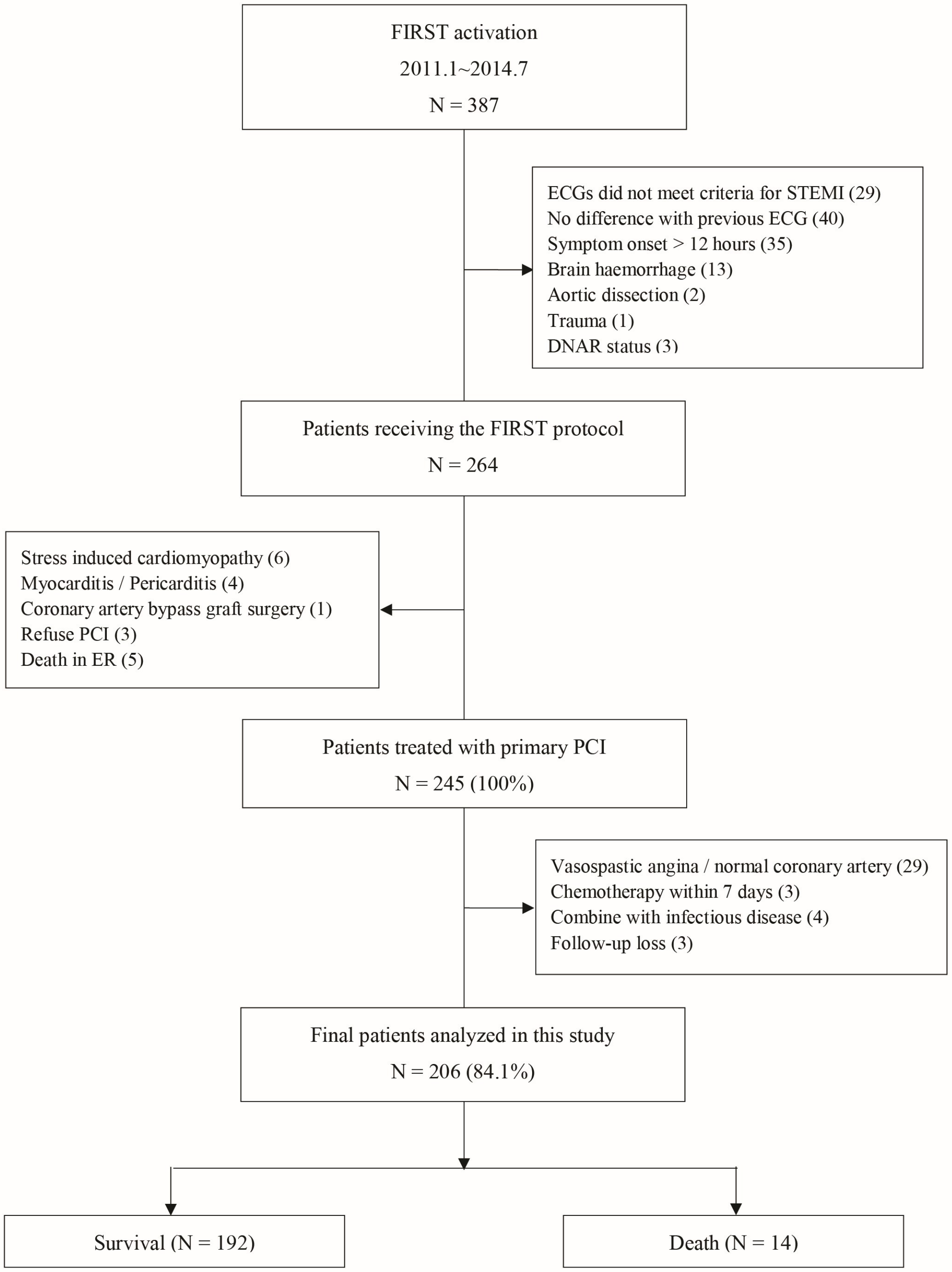

Supplement 4. Flow diagram of the patient enrolment in the external validation cohort

Supplement 5. Inclusion and exclusion of the study patients

Upon arrival of a patient to the emergency department (ED), the physicians, nurses, and emergency medical technicians in the triage area identified candidates for the Fast Interrogation Rule for ST-elevation Myocardial Infarction (FIRST) program as soon as possible according to pre-determined protocols. Preferentially, patients with typical or non-specific symptoms of suspected acute myocardial infarction (e.g., chest pain, epigastric pain, syncope, dizziness, vertigo, shock, dyspnoea, nausea, and/or vomiting) were examined. Simultaneously, a 12-lead electrocardiogram (ECG) was performed in the triage area.[1]

The criteria for critical pathway activation were based on the ECG criteria in the standard STEMI guidelines and the duration since the onset of chest pain.[1] ST-segment elevation in the ECG criteria was defined as J-point elevation on two or more contiguous leads, with a threshold of >2 mm in the precordial leads or >1 mm in other leads, and a new or presumed new left bundle branch block. When a patient had at least one predetermined ECG warning criterion for ST-segment elevation on ED arrival and presented within 12 h of the onset of symptoms, the triage ED physician activated the FIRST program by selecting the activation icon on the order entry window.[1]

We excluded patients lost to follow-up; treated without primary percutaneous coronary intervention (pPCI); with stress-induced cardiomyopathy; with "do not attempt resuscitation" status; or with comorbid conditions such as hematologic malignancy, chronic inflammation, current infection, and use of immunosuppressive agents or chemotherapy within 14 days before ED admission (Fig. 1).

Supplement 6. The delta neutrophil index (DNI)

The DNI was obtained using leukocyte differentials obtained from the two independent channels in the ADVIA hematologic analyser. Regarding polymorphonuclear (PMN) myeloid-derived suppressor cells, which are considered immature neutrophils, the DNI values reflect myeloperoxidase (MPO)-reactive cells lacking nuclear lobularity as PMN myeloid-derived suppressor cells, and may detect circulating immature granulocytes as the leukocyte subfraction.[2 3]

The optical system based on the MPO tungsten-halogen channel measures and differentiates neutrophils, eosinophils, lymphocytes, monocytes, and large unstained cells based on size and MPO staining intensity. Using cluster analysis, the white cells are subsequently identified and counted. Neutrophils are large and positive for peroxidase activity. Monocytes are slightly smaller, with much less peroxidase activity. Lymphocytes are also smaller and exhibit no peroxidase activity. Eosinophils stain intensively for peroxidase. White cells, known as large unstained cells, are large but unstained for peroxidase. These characteristics allow the clear separation of white blood cells and accurate differential counting. The optical system based on the lobularity/nuclear density channel laser-diode can calculate and classify cell types with respect to lobularity/nuclear density and size.[2,3]

The DNI was calculated by subtracting the fraction of mature PMN leukocytes from the sum of the MPO-reactive cells, using the following formula: DNI = (neutrophil subfraction + eosinophil subfraction measured in the MPO channel) − (PMN subfraction measured in the nuclear lobularity channel).[2,3] Currently, only the ADVIA automated blood cell analyser can provide DNI values, because other automated analysers do not use an optical system based on both an MPO tungsten-halogen channel and a lobularity/nuclear density channel.

Supplemental references

1. Park, Y. S. *et al.* Effectiveness of a multidisciplinary critical pathway based on a computerised physician order entry system for ST-segment elevation myocardial infarction management in the emergency department: a retrospective observational study. *BMJ Open* **6,** e011429 (2016)

2. Park, B. H. *et al.* Delta neutrophil index as an early marker of disease severity in critically ill patients with sepsis. *BMC Infect Dis* **11,** 299 (2011).

3. Nahm, C. H., Choi, J. W. & Lee, J. Delta neutrophil index in automated immature granulocyte counts for assessing disease severity of patients with sepsis. *Ann Clin Lab Sci* **38,** 241-246 (2008).
